# Modeling expression quantitative trait loci in data combining ethnic populations

**DOI:** 10.1186/1471-2105-11-111

**Published:** 2010-02-27

**Authors:** Ching-Lin Hsiao, Ie-Bin Lian, Ai-Ru Hsieh, Cathy SJ Fann

**Affiliations:** 1 Division of Biostatistics, Institute & Department of Public Health, National Yang-Ming University, Taipei 112, Taiwan; 2 Department of Mathematics, National Changhua University of Education, Changhua 500, Taiwan; 3 Institute of Biomedical Sciences, Academia Sinica, Taipei 115, Taiwan

## Abstract

**Background:**

Combining data from different ethnic populations in a study can increase efficacy of methods designed to identify expression quantitative trait loci (eQTL) compared to analyzing each population independently. In such studies, however, the genetic diversity of minor allele frequencies among populations has rarely been taken into account. Due to the fact that allele frequency diversity and population-level expression differences are present in populations, a consensus regarding the optimal statistical approach for analysis of eQTL in data combining different populations remains inconclusive.

**Results:**

In this report, we explored the applicability of a constrained two-way model to identify eQTL for combined ethnic data that might contain genetic diversity among ethnic populations. In addition, gene expression differences resulted from ethnic allele frequency diversity between populations were directly estimated and analyzed by the constrained two-way model. Through simulation, we investigated effects of genetic diversity on eQTL identification by examining gene expression data pooled from normal quantile transformation of each population. Using the constrained two-way model to reanalyze data from Caucasians and Asian individuals available from HapMap, a large number of eQTL were identified with similar genetic effects on the gene expression levels in these two populations. Furthermore, 19 single nucleotide polymorphisms with inter-population differences with respect to both genotype frequency and gene expression levels directed by genotypes were identified and reflected a clear distinction between Caucasians and Asian individuals.

**Conclusions:**

This study illustrates the influence of minor allele frequencies on common eQTL identification using either separate or combined population data. Our findings are important for future eQTL studies in which different datasets are combined to increase the power of eQTL identification.

## Background

Several microarray platforms and various statistical methods have been applied in a large number of association studies to identify candidate genes with causative potential. However, only a few studies have provided insight into the functional variant(s) or mechanism(s) underlying these diseases. Single nucleotide polymorphisms (SNPs) are the most common genetic inter-individual differences in the human genome, and through various mechanisms, they can alter the amount of mRNA produced [1]. When individuals are subjected to both DNA sequence polymorphism array genotyping and microarray-based gene expression (GE) profiling, genome-wide joint analysis for identification of expression quantitative trait loci (eQTL) becomes feasible [2]. Several recent investigations have surveyed eQTL using human lymphoblastoid cell lines derived from healthy individuals in single or multiple ethnic populations to generate global regulatory networks in humans [3-7]. In addition, eQTL may have an impact on complex diseases and clinical phenotypes such as obesity and diabetes [8-10].

Data collection for eQTL studies is two dimensional. One dimension examines gene expression levels, which are believed to contribute to phenotypic differences between individuals [11]. The second dimension examines SNP genotypes in which variation in a given population is correlated with disease; most of these variations underlying complex traits are found in regulatory elements of the genome [12]. Therefore, understanding the relationships between transcript abundance and specific genomic markers will likely uncover the molecular basis of phenotypic diversity and improve interpretation of patterns of expression variation in disease [13-15].

Recent studies of HapMap populations have identified numerous eQTL [5-7,16]. However, only a fraction of these eQTL is reproducible across populations; diversity of SNP density between populations represents one probable explanation for the lack of reproducibility [17]. Combining samples across populations may increase sample size and enhance both genetic dissimilarity and the range of variation of GE, thereby increase the statistical significance of identified eQTL [18-20].

Spielman et al. (2007) used an independent approach in which gene expression levels were regressed on SNP genotypes for unrelated CEU (Utah pedigrees of the Centre d'Etude du Polymorphisme Humain), and CHB + JPT (Han Chinese in Beijing and Japanese in Tokyo) samples [5]. Common eQTL were identified when the SNP-GE association was significant in both populations [5]. This procedure may only afford limited detection of common eQTL because the two tests are carried out simultaneously. To increase detection, Stranger et al. (2007) combined the data among populations and used conditional permutations to assess the significance of the SNP-GE associations [17]. Although conditional permutation can be used to manage inflation of the association p-value that is generated from population-level difference, SNP-GE associations in the combining data require appropriate adjustments for possible dissimilar population structure in the model. Veyrieras et al. (2008) combined samples from several populations using the normal Quantile Transformation (QT) method to avoid spurious eQTLs arising from differences in population structure [21]. Unfortunately, this technique may alter SNP-GE associations and mislead conclusions because expression values in each population are forced to have the same distribution. For instance, when gene expression is directly and additively modified by a biallelic SNP (e.g., A, G), those individuals homozygous for the G allele will have high expression levels in comparison with those homozygous for the A allele. If a population has a higher frequency of genotype AA, then the mean expression level of individuals with genotype AA will approach zero after normal quantile transformation. Similarly, if the other population has a higher frequency of genotype GG, the mean expression level of individuals carrying the genotype will also move toward zero. Therefore, the SNP effect will be diminished if the analysis combines the transformed expression values to examine SNP-GE associations. Indeed, the mean expression differences between genotypes will be minimized owing to differences in allele frequency. Although many studies have focused on multi-marker analyses, hot spot identification, and type I errors, the most suitable statistical method for microarray-based eQTL studies comprised of data from more than one population remains unclear.

To address this question, we employ the meta-analysis concept [22], a statistical implementation to synthesize and integrate information across a number of independent studies to estimate the associations between genotype and GE of a sample containing two populations. Meta-analysis detects modest associations and has been used extensively in genome-wide association studies (GWAS) [23]. In the present study, we use a constrained two-way model (CTWM), in which different populations and different genotypes represent the two-way variables, to jointly assess SNP-GE associations in individuals from two populations. This model assumes that the mechanisms of SNP regulation of GE are similar and allows for heterogeneous non-genetic effects on expression between populations. The word "constrained" refers to the constraint that there is no interaction between the two variables in the CTWM. In other words, the SNP effect sizes on GE in populations would be regarded as identical in the CTWM. In addition, we extend the CTWM to divide ethnic GE differences into two parts. The first part represents baseline expression differences, which can be affected by non-genetic factors such as different environmental conditions across populations, and is termed baseline difference (BD). The second part represents quantification of GE differences between populations resulting from genotype frequency differences (genetic), and is termed genetic score (GS). This allows us to examine whether differentially expressed genes between populations are caused by genetic markers or non-genetic factors. This information is unattainable if associations between SNPs and GE are estimated separately for each population.

In the following sections, we briefly describe how common eQTL are identified between two populations using the independent group (IG) method, in which two one-way ANOVA are performed independently. Then, we describe the CTWM for combining two independent unbalanced one-way models to identify eQTL. Using this model, the magnitude of GE differences between populations resulting from genotype frequency diversity can be directly estimated. Furthermore, we use the GS obtained from CTWM to identify putative functional SNPs, and is termed CTWM-GS method. In the simulation study, we control allele frequency differences between populations to compare the power of different methods for identifying eQTL. Finally, we apply our method to some authentic datasets to identify gene regulation mechanisms, examine the assumptions of homogeneous regulation mechanisms, and identify additive inheritance patterns and SNPs that induce GE differences between CEU and Asian (CHB + JPT) cohorts.

## Methods

In the present study, we use conventional gene-base mapping, such as the common technique of mapping eQTL to determine gene expression traits one gene at a time. Consider *n *unrelated individuals observed from two different ethnic populations, *i *= 0, 1. Log_2_-transformed gene expression values for the *k*th replication on the *j*th genotype in the *i*th population, denoted by *y*_*ijk *_(where *i *= 0, 1; *j *= 0, 1, 2, representing the number of minor alleles carried; *k *= 1,...,*n*_*ij*_; Σ_*j *_*n*_*ij *_= *n*_*i*_) are used to reduce the impact of outliers and to ensure normality. The gene expression values, *y*_*ijk *_(for *k *= 1,...,*n*_*ij*_), are assumed to be independently distributed as N(*μ*_*ij*_, *σ*^2^). Although the additive model has been frequently used in eQTL studies, many variations in transcription levels cannot be explained by this model [24,25]. In comparison, co-dominant genetic models, in which three genotype effects are estimated, have good overall performance for identification of any simple inheritance patterns in QTL mapping [26], and is therefore being employed in the following analysis.

### The independent group (IG) method

Because of possible heterogeneity between populations, many studies analyze SNP-GE associations independently for each ethnic population and summarize their results to identify common eQTL [5,6,27,28]. Without loss of generality, we assume that three genotypes are observed for a biallelic SNP in each population. In addition, we assign a common genotype *c *(where *c *= *argmax*_*j *= 0, 1, 2 _Π_*i *= 0, 1 _*P*_*ij*_, and *P*_*ij *_is the proportion of individuals carrying genotype *j *in the *i*th population) between populations.

The cell mean model has been used extensively for unbalanced data in which the number of observations varies from one genotype to another. Based on the cell means, within the *i*th population, the *n*_*i *_signals in a given gene are decomposed into:

*y*_*ijk *_= *μ*_*ij *_+ *e*_*ijk*_, where *μ*_*ij *_is the cell mean, and *e*_*ijk *_is assumed to be independently distributed as N(0, *σ*^2^). If the common genotype *c *is assumed to be 0, this model can be rewritten using another parameterization as *y*_*ijk *_= *μ*_*i*0 _+ *τ*_*ij *_+ *e*_*ijk*_, where *μ*_*i*0 _is the expression baseline in the *i*th population; *τ*_*ij *_= *μ*_*ij *_- *μ*_*i*0 _when j ≠ 0, which represents the GE difference between individuals with genotype *j *and genotype 0. This reparameterization model is used in the subsequent analysis.

The goal of the IG analysis is to test the null hypothesis *H*_0_: *τ*_*i*1 _= *τ*_*i*2 _= 0 in each population. It is straightforward to observe maximum likelihood estimators through a normal equation. The estimators of *μ*_*i*0 _and *τ*_*ij *_are  and ,

where , and the *F*-statistic calculated from ANOVA can be used to test the null hypothesis. In the IG analysis, the parameters are estimated separately in each population. This approach is free of model assumption and allows for heterogeneity of SNP effects (*τ*_*ij*_) between populations. A common eQT locus is then identified if the two hypotheses *H*_0_: *τ*_01 _= *τ*_02 _= 0 and *H*_0_: *τ*_11 _= *τ*_12 _= 0 are simultaneously rejected.

### The QT method

Although the power of eQTL identification is influenced by the magnitude of the SNP effect on gene expression, it is clear that magnitude of test statistics may vary as a function of sample size and allele frequency, even if the SNP effects in the two populations are identical. On the other hand, merging data set with proper adjustment before statistical testing may enrich data variation and is more effective for detection of common eQTLs compared with the previous method. To avoid spurious eQTLs in the data combined from multiple populations due to population structure, Veyrieras et al. (2008) applied a normal quantile transformation (QT) to each gene, within each population before combining data [21] (for details, see additional file 1: Supplementary material).

### The constrained two-way model (CTWM) method

We use a CTWM strategy to identify common eQTLs between populations. Because the results from the IG method are to be compared with those obtained from combined data, we consider *c *= 0 as the baseline genotype, and individuals with the common genotype (*j *= 0) in each population are assigned a different GE mean value, *μ*_*i*0 _for *i *= 0, 1, to allow for heterogeneity of GE baseline. We assume homogeneity of SNP regulatory mechanisms across ethnicity by defining *E*(*y*_*ijk *_- *y*_*i0k*_) = *τ*_•*j *_for all *j *= 1, 2 and *i *= 0, 1. Consequently, the *n *(where *n *= *n*_*0 *_+ *n*_*1*_) signals can be expressed as *y*_*ijk *_= *μ*_*i*0 _+ *τ*_•*j *_+ *e*_*ijk*_, where the *e*_*ijk*_'s are assumed to be independently distributed as N(0, *σ*^2^). Because the two independent unbalanced one-way models in the IG approach are combined into a CTWM, the analysis of SNP-GE associations can be performed by testing the null hypothesis *H*_0_: *τ*_•1 _= *τ*_•2 _= 0 using the partial *F*-statistic:

, where B is in the matrix form , *q *is the rank of the matrix B, X is the design matrix, *SSE *represents the error sum of squares, and *v *represents the degree of freedom for the *SSE *(for details, see additional file 1: Supplementary material).

### The CTWM-GS method

The ultimate goal in the present genetic genomic study, which incorporates data based on two phenotypes, is to identify not only the association between SNPs and GE but also the relationship between SNPs and phenotypes. In general, investigators aim to identify SNPs that are associated with particular GE profiles and discern whether the difference of allelic frequency of a particular SNP induces a gene expression difference between populations, termed eSNP [29]. Using the CTWM described above, the gene expression differences between populations can be directly partitioned into two parts (genetic differences, i.e., GS, and non-genetic differences, i.e., BD) and independently tested.

BD, by previous definition, is the GE baseline difference between populations that could be parameterized as *μ*_00 _- *μ*_10_. Similarly, GS, which is the GE difference resulted by the genotype frequency differences between two populations could be represented as (*P*_01 _- *P*_11_) *τ*_•1 _+ (*P*_02 _- *P*_12_) *τ*_•2_. We solve the normal equation of the CTWM by maximum likelihood estimation and express the estimators using the cell mean (see additional file 1: Supplementary material). Consequently, the estimators of GS and BD can be expressed as follows:

where *M*_*j *_= *n*_0*j *_*n*_*ij*_; *A*_*j *_= *n*_o*j *_+ *n*_1*j*_; ; *s *= {0,1}\{i}, which means *s *is an element in {0,1} but not in {i}, and *r *= {0,1,2}\{ j, t}, which means *r *is an element in {0,1,2} but not in {j, t}.

These two estimators indicate that BD and GS are invariant with respect to the baseline genotype chosen, since they are free of index *j*. Because we observe a unique solution for both BD and GS, these estimates can be tested using the null hypotheses *H*_0_: *μ*_00 _= *μ*_10 _and *H*_0_: (*P*_01 _- *P*_11_) *τ*_•1 _+ (*P*_02 _- *P*_12_) *τ*_•2 _= 0 via the partial *F*-statistic described in the CTWM method with *B *= (1, - 1, 0, 0) when testing BD and *B *= (0, 0, (*P*_01 _- *P*_11_), (*P*_02 _- *P*_12_))when testing GS, respectively. If the null hypothesis for GS is rejected, then the SNP-GE association will be claimed as an eSNP.

In addition, these two quantities can be represented as two non-overlapping scores and separated from the arithmetic mean difference between populations as below:

### Simulations

To demonstrate the performance of the models for common eQTL identification, we carried out simulations using varied parameters consisting of allele frequency of population 0 (*P*_*0*_), baseline (D) and allele frequency (d) differences between two populations and the magnitude of the SNP effect on GE (E). In these simulations, we compared both the Type I error rate and power of eQTL identification among three different common eQTL identification methods including IG, CTWM and QT. An additional CTWM-GS in identifying eSNPs was compared for various allele frequency differences. We further examined the precision and accuracy of the estimates (*τ*_•1_) for the CTWM and QT method, and BD and GS for the CTWM-GS. In scenario 1, gene expression data were simulated with differential expression resulting only from allele frequency differences between two populations. In scenario 2, the simulation was performed with differential expression resulting from both population-level and genetic-level differences to determine whether genetic factors can be examined independent of non-genetic factors (for details, see additional file 1: Supplementary material).

### Ethnic population data: HapMap

To validate the performance of eQTL identification models for GWAS data, we reanalyzed the gene expression dataset from Gene Expression Omnibus (GSE6536) using 60 unrelated CEU and 90 CHB + JPT individuals. Recent studies have demonstrated that probe sequences including SNPs would influence the hybridization on microarrays and cause false *cis *eQTL [30]. The rationale for this effect is that the mRNA with an identical sequence as the probe designed would hybridize better. To avoid false positives of such eQTL, the subset of 14,456 expression traits excluding probes that had polymorphisms in the probe-target sequence, as was reported in Stranger et al.'s study [17], was thus used in the analysis. Remapping of traits was performed using Bioconductor software [31] with the illuminaHumanv1.db package [32]; 4,259 expression traits lost their chromosome position information and were excluded in the subsequent analysis. The corresponding Affymetrix 500 K genotype expression data were downloaded from the Affymetrix website http://www.affymetrix.com and SNP genotypes of each individual were called based on the Affymetrix BRLMM algorithm. The established imputation method 'beagle' was used to impute missing genotypes [33].

For each expression trait, SNP-GE associations were examined by each of the IG, CTWM and CTWM-GS methods, provided that both the SNP and the gene were on the same chromosome. Our previous results showed that the QT method was highly influenced by allele frequency differences between populations, the comparisons between the QT method and others would thus be difficult to interpret. Therefore the QT method was not included in the subsequent real data analysis. In the following analysis, a conservative threshold with a *p*-value < 10^-6 ^was used to identify the eQT locus for each trait. This threshold yielded a false discovery rate (FDR) [34] of 0.85% and 1% for the CTWM and the CTWM-GS method, respectively, when 10,197 expression traits were analyzed. For expression traits having more than one significant eQT locus, the SNP with the smallest association p-value was selected as the putative eQT locus. In the following analysis, *local *is defined as the segment of the chromosome spanning from 100 kb upstream to 100 kb downstream of the gene sequence of interest, and *distant *is defined as the region outside of *local *on the same chromosome.

For significant eQTL identified by the CTWM, full- and reduced-model strategies were performed to evaluate the heterogeneous expression baseline, homogeneous SNP effects between populations, and the assumption of non-additive. After examining the applicability of the CTWM, we used the CTWM-GS method to investigate whether differential gene expression between populations is affected by genetic or non-genetic factors. Furthermore, we evaluated the contributions of functional SNPs identified by the CTWM-GS method, to GE differences between the CEU and Asian cohorts using the hierarchical clustering method. The analyses were performed on the R package http://www.r-project.org.

## Results

### Simulations: Comparing the probabilities of rejecting null hypotheses

**Figure 1:**
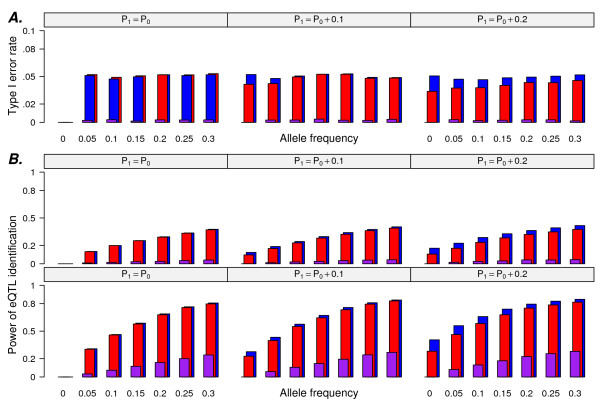
**Simulations without inclusion of baseline differences I**. The allele frequency of population 0 (*P*_*0*_) was analyzed with respect to (A) type I error rate (with *D *= 0 for d = 0, 0.1, 0.2) or (B) power of eQTL identification; upper and lower panels represent E = 0.3 and 0.5, respectively. The three color bars represent the three different testing methods as follows: purple, IG; red, QT; blue, CTWM.

We compared the observed Type I error rate () among three eQTL identification methods (IG, QT, CTWM) with different *d *values by setting *E *equal to zero (Figure 1A). Because two tests are carried out simultaneously in the IG method, it comes as no surprise that this method yielded the lowest  for each simulation. At a fixed Type I error rate of 0.05,  for the QT method conforms to this restriction only in the case of *d *= 0 (Figure 1A, *d *= 0). For other cases,  of the QT method was affected by the allele frequency difference between populations; e.g.,  decreased as the *d *value increased under the same *P*_*0 *_(Figure 1A, *d *= 0.1 and 0.2). For both *d *= 0.1 and 0.2, the lowest error rate was observed when *P*_*0 *_was less than 0.05. This implicates that QT method is unsatisfactory when simulating SNPs that are polymorphic only in one population. In contrast, using the CTWM,  was stable for all cases simulated.

**Figure 2:**
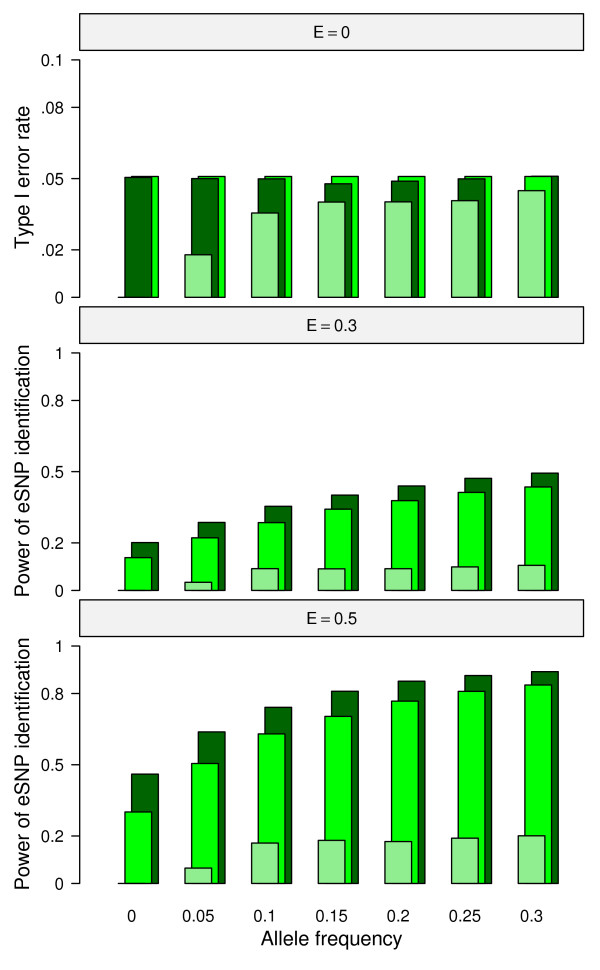
**Simulations without inclusion of baseline differences II**. The allele frequency of group 0 (*P*_*0*_) was analyzed with respect to type I error rate (upper panel, E = 0) and power (middle panel, E = 0.3; lower panel, E = 0.5) of eSNP identification, respectively. The three color bars represent the three different allele frequency differences as follows: light green, d = 0; green, d = 0.1; dark green, d = 0.2.

For the CTWM-GS method, which incorporated allele frequency differences between populations to identify eSNPs,  was stable for all cases simulated except for the case of *d *= 0 (Figure 2, *E *= 0). Because for each SNP simulated, the genotype frequencies were assumed to follow Hardy-Weinberg Equilibrium, it was possible that simulated genotype frequency differences drifted a bit away from zero even with a fixed d = 0. If the genotype frequency differences were to be fixed at zero, the estimates of GS would equal to zero and  of the CTWM-GS method would therefore equal to zero simultaneously.

The following describes simulations in which differential gene expression was allowed only as a result of allele frequency differences between two populations. For all methods, we compared the power of eQTL identification with *E *equal to 0.3 and 0.5 and *d *varying as 0, 0.1, or 0.2 with each *E *value. As above, the IG method yielded the lowest power to detect common eQTL, and its utility was limited by the population with the lowest allele frequency; the curves representing the power of eQTL identification were very similar in the cases of *d *= 0, 0.1, and 0.2 under the same *E *value (Figure 1B). When allele frequency was the same in two populations, the QT and CTWM methods had the same ability to detect significant associations at *E *= 0.3 or 0.5 (Figure 1B, *d *= 0). When *d *value varied from zero, however, CTWM outperformed the QT method, and the differences of power in eQTL identification between these two methods increased as a function of *d *under the same *E *value (Figure 1B; *d *= 0.1 and 0.2). When CTWM-GS was used to detect eSNP, the power of eSNPs identification was a function of the allele frequency differences between populations (*d*) under same *E *and *P*_*0 *_(Figure 2, E = 0.3, 0.5); i.e., when the magnitude of the SNP effect and allele frequency of population 0 are fixed, SNP-GE associations having a larger difference in allele frequency between populations were easier to be detected by the CTWM-GS method.

Simulations were also performed with *D *= 1, and results showed that  and power curves of eQTL and eSNPs identification were very similar to those obtained when *D *= 0 (see additional file 2: Simulations with baseline differences).

### Simulations: Comparing the estimates

**Figure 3:**
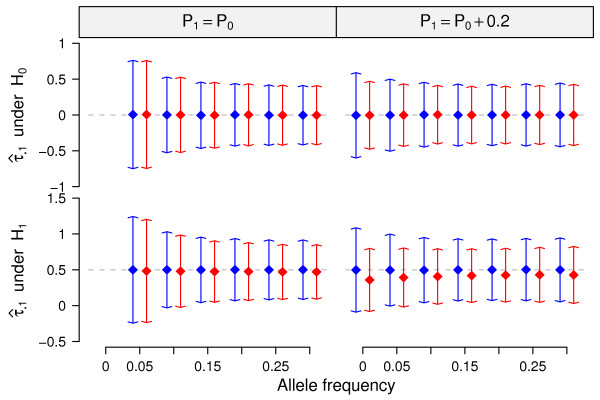
**Estimates in simulations**. The dots are means of the  estimated by CTWM (blue) and QT (red) methods under null (upper panel, E = 0) and alternative (lower panel, E = 0.5) hypotheses. Arrows of each dot represent the 95% confidence interval calculated from 10,000 simulations. Dash lines are the true values of *τ*_•1 _used in the simulations.

We examined precision and accuracy of the estimates (*τ*_•1_) for the CTWM and QT methods under the null (i.e no SNP effect on GE, *E *= 0) and alternative (i.e *E *= 0.5) hypotheses. The simulation results showed that, under the null hypothesis, both of these two methods could estimate *τ*_•1 _accurately; i.e., average of the estimates fit the expected value given the simulation (Figure 3). The QT method provided slightly higher precision than CTWM when *P*_*1 *_was larger than *P*_*0*_, especially when allele frequencies were small (Figure 3). Under the alternative hypothesis, however, the estimates of the QT method were influenced by the *d *value; e.g., if *d *and *E *were fixed at 0.2 and 0.5 respectively, the QT method somewhat underestimated *E *value (Figure 3). In contrast, the CTWM could accurately estimate *E *value and was not influenced by allele frequency differences between populations for all scenarios.

We also explored the estimates of GS and BD in CTWM-GS method with *D *= 1. Under the null hypothesis, estimations of the GS and BD parameters were reliable despite the fact that GS variations were affected by the allele frequency. Similar results were observed under the alternative hypothesis (see additional file 3: Estimates of BD and GS).

### Analysis of HapMap populations using the IG method

**Figure 4:**
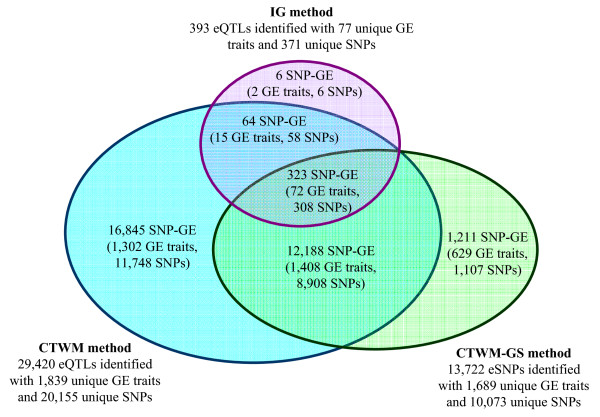
**Summary of SNP-GE data generated using the IG, CTWM and CTWM -GS methods**.

Using a pair of HapMap population data sets, 349 (3.4%) expression traits were mapped to significant eQTL either in the CEU or Asian population by the IG method; however, only 77 expression traits with 393 eQTL were common between the two populations (Figure 4). When examining the effects of ethnic-genotype interactions on gene expression among the 77 traits with common eQTL by both the full and reduced two-way ANOVA models (the full model contains ethnic-genotype interaction term, and the reduced model does not), only 11 (14.3%) putative eQTL showed significant interaction effect at a threshold of q-value < 0.05 [35]. Further investigation of the positions of the 77 putative eQTL revealed that 61 resided in the *local *region. These results indicated that the IG method was able to identify common eQTL near or in the target genes of interest and that for most eQTL the SNP effects on gene expression levels were similar in populations. To clarify, in the subsequent analysis, all of the FDR calculations were based on Storey's q-value approach [35], which is considered to be more powerful than other FDR approaches. However, in the whole eQTL study across all SNP-GE association tests, the prodigious number of p-values (~2.5 × 10^9^) made it computationally prohibitive to run Storey's q-value, so Benjamini and Hochberg's FDR approach [34] was used instead.

### Analysis of HapMap populations using the CTWM method

Using CTWM, 1839 (18%) expression traits had at least one SNP with a statistically significant SNP-GE association (Figure 4). Notably, except for six IG-specific common eQTL, all 393 significant SNP-GE associations identified by the IG method were also identified by CTWM (Figure 4), although the IG method allowed for heterogeneous SNP effects between groups. For the six IG-specific eQTL, the p-values of the ethnic-genotype interactions evaluated using the *F*-statistic ranged from 1.08 × 10^-16 ^to 1.52 × 10^-9^; the CTWM was therefore not able to detect significant associations for these six eQTL. One of the possible reasons is that, when there are differences in the magnitude of the SNP effect on GE, particularly for SNPs that have opposite effects in the two populations, CTWM may fail to detect SNP-GE associations. Nevertheless, for two of the expression traits with IG-specific eQTL, CTWM identified other SNPs with strong SNP-GE associations. For example, gene *S100A8*, detected using expression probe GI_21614543-S, was mapped to the putative eQTL rs3896232 with an association p-value of 1 × 10^-16^. Thus, the set of 77 expression traits with at least one eQT locus detected by the IG method was a subset of the 1839 expression traits detected by CTWM.

### Analysis of HapMap populations: *local *and *distant *effects

Among the 1839 putative eQTL identified by the CTWM, 438 (24%) were identified as *local *eQTL and 1401 (76%) were *distant *eQTL. In addition, average of the -log_10 _association p-values were 12.3 and 9.7 for the *local *and *distant *putative eQTL, respectively, and the difference between these two means was significant (*p *= 1.5 × 10^-6 ^by Welch's t-test) (see additional file 4: Summary of putative eQTL generated using CTWM). This result suggests that eQTL near target genes have smaller p-values than those further away from genes as identified by CTWM. Examination of the minor allele frequency (MAF) for eQTL with respect to chromosomal position showed that the MAF for *local *and *distant *putative eQTL in the combined population were 0.28 and 0.1, respectively. The difference of these two frequencies was significant (*p *< 1 × 10^-16 ^by Welch's t-test) (see additional file 4).

### Analysis of HapMap populations: justification of model assumptions

We used full- and reduced-model strategies to justify the assumption that the magnitude of the SNP effect is homogeneous by examining the genotype-ethnic interaction term in the model. Of the 1839 SNP-GE pairs identified by the CTWM, only 44 (2.4%) putative eQTL had significant genotype-ethnic interaction following the FDR correction (q-value < 0.05). In contrast, when we investigated the heterozygous non-genetic SNP effect assumption using full- and reduced-model strategies (the full model was fitted to different expression baselines whereas the reduced model was fitted to an identical baseline between populations) for each of the 1839 SNP-GE pairs, there were 1298 (70.6%) traits with a q-value < 0.05.

For genome-wide identification of eQTL, researchers often employ the additive genetic model as a basic inheritance pattern to assess the association between SNP genotype and GE. Using the full- and reduced-model strategies again (the full model using a co-dominant assumption whereas the reduced model using an additive assumption), 874 (48%) out of 1839 putative eQTL were significantly associated with co-dominant assumption (q < 0.05). Among the 874 eQTL, only 49 were in *local *regions. The result suggests that the additive genetic model assumption is applicable to most *local *eQTL, and co-dominant genetic model assumption seems to be more suitable to *distant *eQTL. Further research is needed to confirm these results.

### Analysis of HapMap populations using the CTWM-GS method

The ultimate goal of microarray studies is to generate a list of gene variants for further investigation. Such studies generally involve comparisons of two conditions within a single population or of two clinically distinct populations. We incorporated SNP diversity information from two ethnic cohorts to identify regions of the genome where genetic diversity is associated with gene expression differences between populations.

We have demonstrated the applicability of CTWM for combined populations. Thus, under CTWM, GS was substituted for SNP-GE associations to incorporate SNP diversity information into the analysis. This generated 1689 (16.5%) expression traits that included at least one SNP with a significant GS (threshold p-value < 10^-6^) (Figure 4). Out of these 1689 putative eSNPs, 914 (54%) were included in the set of 1839 putative eQTL identified by CTWM. A high number of intersecting eQTL was expected because both methods explored the random aspect of the SNP effect.

### Analysis of HapMap populations: genetic and non-genetic effects

**Figure 5:**
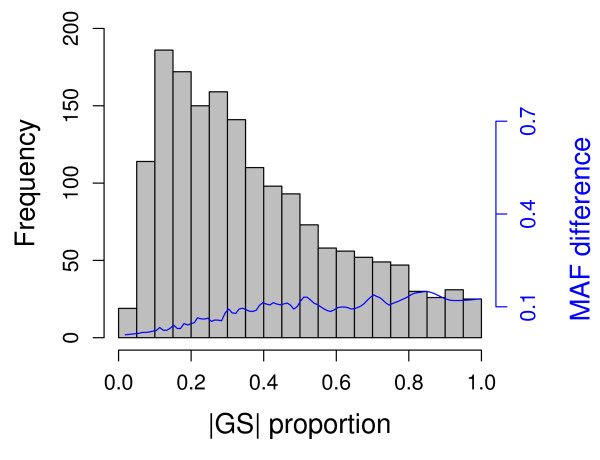
**Summary of Genetic Score analysis**. |GS| proportion was calculated by |GS|/(|BD| + |GS|) as shown in the x-axis. The histogram is a representation of |GS| proportion frequencies (indicated on the left-axis in black) underlying 1,689 SNP-GE pairs identified by CTWM-GS method. The blue line represents the smooth correlation between |GS| proportion and MAF (minor allele frequency) differences (indicated on the right-axis in blue) estimated by the Lowess method underlying the same 1,689 SNP-GE pairs.

Because the CTWM method can partition mean expression differences, this allows for investigation on whether differentially expressed genes are affected by genetic or non-genetic factors. To avoid unnecessary cancellation, we calculated the proportion of genetic factor by |GS|/(|BD| + |GS|). If the denominator was considered as the total expression bias for a particular SNP-GS pair, Figure 5 showed that most of the eSNPs resulted in less than 40% expression biases of their target genes between the CEU and Asian cohorts (Figure 5). Furthermore, we examined the association between these proportions and the MAF differences using a locally weighted scatterplot smoothing (LOWESS) [36]. These results showed that the SNPs with higher proportions were those with greater allele frequency differences (Figure 5). Although the CTWM-GS method still requires the CTWM to test the associations, it provides immediate insight into SNP effects on the variation of GE among populations.

Among the 1689 putative eSNPs detected using the CTWM-GS method, there were 22 SNP-GE pairs represented by 20 unique gene symbols and 19 unique SNPs selected using a GS threshold of 0.5 (|GS|≧0.5) (see additional file 5: Supplementary table). These eSNPs contribute to more than 40% of the expression bias and have greater MAF differences between populations, except those genes with higher BD values. Most of the 22 pairs had significant SNP-GE association in at least one population tested by using IG method; the non-significant associations are likely to result from a low MAF. For instance, the SNPs rs1419772, rs2337387 and rs604127 have minor allele frequencies ranging from 0.01 to 0.06, and their corresponding SNP-GE association p-values range from 0.07 to 0.7 in the Asian cohort (see additional file 5: Supplementary table). Moreover, the genes *C3orf14 *and *C8orf13 *lacked sufficient evidence to be considered as differentially expressed in the case where we compared their expression levels directly and omitted the eSNP information (see additional file 5: Supplementary table). However, these seemingly similar expression levels are due to opposing effects of genetic and non-genetic factors on these genes in the CTWM. Therefore, it is highly important that differences in allele frequency between populations be included in analyses of inter-population differential gene expression.

### Analysis of HapMap populations: applying the GS to population studies

**Figure 6:**
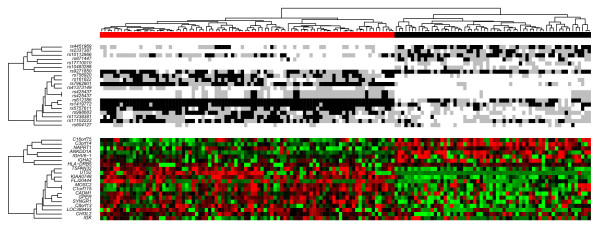
**Heatmap of hierarchical clustering**. The vertical hierarchical cluster shows that the CEU (black) and Asian (red) populations can be separated by clustering their genotypes as shown in the horizontal hierarchical tree. Upper heatmap: black shading represents individuals homozygous for the upregulated allele, and gray shading represents heterozygous. White shading indicates individuals homozygous for the downregulated allele. The lower heatmap represents expression of the genes corresponding to the eSNPs in the upper heatmap; intensity of red is proportional to degree of expression above the mean, and intensity of green is proportional to degree of expression below the mean.

To evaluate the genetic contributions to the observed differences in gene expression between the CEU and Asian populations, we first clustered all individuals by genotype relative to the 19 unique eSNPs identified using a GS threshold of 0.5 (|GS|≧0.5). For each eSNP, individuals homozygous or heterozygous for the upregulated allele or homozygous for the downregulated allele were assigned values of 2, 1 or 0 respectively. When the Spearman rank correlation coefficient was used to measure the similarity between individuals, the CEU and Asian cohorts were separated into two distinct groups (Figure 6). The cluster also divided SNPs into two groups, where group 1 was comprised of seven SNPs from alleles associated with upregulated expression that are mostly found in the CEU cohort in contrast to the Asian cohort. Group 2 consisted of 12 SNPs from alleles associated with upregulated expression that are mostly found in the Asian cohort compared to the CEU cohort. When the cluster order was fixed, the corresponding expression pattern was visualized using an expression heatmap. To avoid bias from non-genetic factor differences, we adjusted the population-level expression by adding the estimated BD score to each CEU individual for each expression trait. Similar to genotype clustering, the expression pattern was generally divided into two groups corresponding to regulatory SNPs, and the majority of the individuals with genotype assignments of 2 or 1 had higher gene expression levels (Figure 6).

## Discussion

Taken together, our simulation studies demonstrate that the IG method is unsatisfactory for identifying common eQTL. Although this method is robust and does not require model assumptions, the power of this method is poor even when the magnitude of the SNP effect is large. On the other hand, we demonstrate that if two population data are combined prior to statistical testing, CTWM outperforms the QT method and can manage the bias arising from allele frequency differences. In particular, if a study is focused on investigating genetic differences between groups, CTWM-GS method can be used regardless any possible population-level differences. These results indicate that the CTWM and CTWM-GS methods are effective for eQTL and eSNP identification, respectively, regardless of baseline differences between groups, and that these methods have the potential to distinguish population-level and genetic-level differences between groups. Note that although the nominal level of significance is fixed (say, at 0.05), the empirical type I error rates do not always remained fixed. In the simulations, the type I error rate could be inflated as the trade-off for higher power. Therefore, when comparing different methods, type I error and power should be considered simultaneously.

In current eQTL studies, IG method is a conventional technique to identify the significant eQTL commonly presented in two populations [5,28]. The composite hypothesis is a union of 2 sub-hypotheses in each population, and it will be rejected only if both 2 sub-hypotheses are rejected simultaneously. The low type I error and power are expected. In addition, we have used IG method with the hypothesis composed of the intersection of 2 sub-hypotheses. The hypothesis would therefore be rejected if either of the sub-hypotheses is rejected. Our simulation results showed that the power of this method is still lower (about 25% to 40% lower) than that obtained from CTWM (see additional file 6: Supplementary simulation results). Possible reasons of these results include the smaller sample size due to splitting data, the higher significant threshold due to multiple test adjustment, etc. In addition to CTWM, a two-way ANOVA with an interaction term is a comprehensive model that allows for different SNP effects in each population. The simulation results based on this method still showed lower power in detecting SNP effects on gene expressions compared to CTWM, except when *P*_*0 *_was zero (see additional file 6: Supplementary simulation results). That is because the number of parameters used in the two-way ANOVA with interactions was the same with that in CTWM when *P*_*0 *_was zero.

Using the F distribution to test GS directly, our simulations showed that if the null hypothesis (H_0_: GS = 0) was under the assumption of E = 0, then the probabilities of rejecting the H_0 _were properly controlled around 0.05 given level *α *= 0.05 (Figure 2; E = 0). When a more general null hypothesis for GS = 0 was considered as the condition of E = 0 or d = 0, however, the rejection probability under d = 0 was inflated as E increased; e.g. the rejection probability reached 0.2 given level *α *= 0.05, when E = 0.5 and d = 0 (Figure 2). This deviation was possibly due to the fact that the simulated difference of genotype frequency might be drifted away from zero even when d was fixed at 0. These estimated differences of genotype frequency between populations had been regarded as constants in the test statistic and therefore higher E values produced higher rejection probabilities for H_0_. Additional permutation tests were performed to generate empirical null distributions of the test statistic and compare the rejection probabilities with those observed from using the F distribution directly. The results showed that permutation approach could partially decrease the rejection probabilities, but the probabilities would still be inflated as E value increased given that d = 0 (see additional file 7: Comparing F test with permutation test). Since those *P*_*ij*_'s in the vector B = (0, 0, (*P*_*01 *_- *P*_*11*_), (*P*_*02 *_- *P*_*12*_)) for testing GS need to be estimated from the data, a further study is needed to discuss the impact of non-constant issue of B.

Using CTWM, we identified 29,420 common eQTL with 1,839 expression traits with the underlying FDR less than 1%, and these traits included all the 77 traits identified by the IG method. These results suggest that CTWM has greater power to identify common eQTL and empirically supports the assumption of homogeneous SNP effects between populations for common eQTL. However, one concern with these reports is that, the use of all SNPs on the array to model eQTL might inflate the number of eQTL and reduce FDR due to redundancy in the SNPs that were not taken into account. We therefore performed a data analysis on the 163,448 tag SNPs obtained from Affymetrix 500 K array using HapBlock program [37]. Results showed that the number of the expression traits with at least one significant eQTL (with p-value < 3×10^-6^) was similar compared to that obtained from using all SNPs on the Affymetrix 500 K array (~1,800 GE traits). However, the number of eQTL identified and SNP mapped were sharply decreased (decreasing from 29,420 to 9,813 for eQTL; from 20,155 to 6,599 for SNPs). We noted that the number of tag SNPs was only a third of the number of SNPs on the 500 K array and about a third of the eQTL were identified (see additional file 8: Results from using tag SNPs). These results suggested that the influence of redundancy in the SNPs was minor in this study.

Further investigation of the mapping positions of the 1,839 putative eQTL revealed that most of the putative eQTL in *local *regions had smaller p-values than those in *distant *regions. This is because *local *eQTL are highly heritable [38] and impact genes more directly in the regulatory sequences. This finding is in concordance with previous *cis *eQTL studies [18,39], however, the majority of the gene expression traits were most significantly associated with eQTL in *distant *regions. This phenomenon could be explained partially by the criterion that we defined as 'most significant'; that is, for a GE trait with multiple significant eQTL, only the SNP with the smallest SNP-GE association p-value was selected into the analysis. In addition, this may also have arisen because gene correlations are inherent in the same biochemical pathway [40] or because of genes that are tightly regulated by hotspots [41]. These possibilities should be investigated further.

Dissecting allele frequencies of the putative eQTL showed that the eQTL at greater distances from the target genes had smaller allele frequencies than those in or near the genes. A number of genetical genomic studies have shown that *cis *eQTL were more reproducible and were usually mapped with higher statistical significance than those in *trans *eQTL [42,43]. Diversified factors underlying the complexity of gene regulation could contribute to this phenomenon, such as polygenic regulation, environmental input and possibly interactions among loci [13,25]. For example, in yeast studies, it has been observed that distinct trans-acting loci could exert contrasting effects on gene expression and highly heritable transcripts could exhibit transgressive segregation or epistatic effects [44]. Therefore, our finding does not necessarily imply causality between the allele frequencies and the type of eQTL. However, rare minor alleles may partially account for the incapability of the IG method to identify common *distant *eQTL. That is, because the samples were separated into two independent studies, common *distant *eQTL would then be difficult to be detected and reproduced in two populations with small sample size and/or small allele frequency. These results suggest that proper combination of different ethnic datasets to increase the sample size can overcome statistical barriers inherent in unbalanced genotype data and might result in an association mapping that is more precise for identification of eQTL.

In the analysis presented, we limited the number of association tests by only considering SNPs on the same chromosome with the gene transcripts, so that only within-chromosome associations were tested. The reason was two fold. First, computation time was extremely long if all chromosomes were considered. Second, a higher threshold was needed given a huge number of association tests performed and would cause lower power in the study with a small sample size. Although the trans-acting eQTL could locate on a different chromosome from where the gene is located [45], recent studies have demonstrated that most of the intense significant signals appeared in the *cis *region by using a loose definition [18,38,45]. Therefore, we only considered mapping the associations by a chromosome-wide strategy. The implications of our results are therefore limited to the same chromosome. However, it provides a crude picture about the characteristics of local- and distant-acting eQTL.

As many other methods, CTWM also assumes that SNP effects are similar between populations in the combined data. This assumption presumably can largely reduce the complexity of the model and the number of parameters needed. After comparing the associations found among all populations, Veyrieras et al. (2008) suggested that significant SNP-GE associations were usually shared in different populations [21]. However, whether the magnitude of the SNP effect on GE was similar for any particular SNP-GE association in different populations remained unclear. Our results indicate that, for the 1,839 putative common eQTL identified by CTWM, only 2.4% had significant interaction effects following FDR correction (q-value < 0.05). This proportion was probably underestimated due to the rigorous inclusion criterion on the 1,839 putative eQTL. Therefore, similar analyses were performed using all 29,420 significant eQTL, and results showed that 2,968 (10%) eQTL had significant signals (q-value < 0.05) for interaction terms. The discrepancy between the two results could be due to high false positives or violations of homogenous SNP effect sizes. Because we restricted our attention to only eQTL identified by CTWM, our results did not imply that all SNP effects were necessarily homogeneous in the eQTL study in multiple populations. Our results exhibited the characters of putative eQTL identified by the model. That is, population-level differences in GE substantially affected eQTL identification, and SNP effects were similar between populations. These results may form a basis for further studies in which different datasets are to be combined to increase the statistical power for identifying common eQTL with similar effects.

In microarray-based GWAS, mapping regulatory variations on gene expression data is more promising than mapping regulatory variations on complex clinical phenotypes [24,46]. By using combined data and CTWM-GS, our goal is to detect associations between SNP and GE and further explore whether the discrepancy between allele frequencies causes the different levels of GE in different populations. It is possible to compare allelic frequencies of SNPs between populations and then test the association of these SNPs with GE data. However, the association between GE and populations can not be directly implied given associations between populations and SNPs which are correlated with GE [10]. For example, a transcript *C8orf13 *was associated with a putative eQTL 'rs998683' (*p-*value < 5 × 10^-14 ^tested by CTWM) and showed high discrepancy in allelic frequencies (*p*-value < 10^-9 ^tested by chi-square) between CEU and Asian cohorts. However, the overall expression level of that transcript was non-differentially expressed between the two cohorts as shown in the additional file 5. This phenomenon was in agreement with the previous genetic genomic study of the childhood asthma, where Moffatt et al. (2007) found the expression level of *ORMDL3 *to be strongly associated with a disease-associated marker 'rs7216389'. However, the overall GE difference between non-asthmatics and asthmatics cohorts was not significant. The advantage of using CTWM-GS is that gene expression differences between populations can be partitioned into two parts--genetic differences (i.e., GS) and non-genetic differences (i.e., BD)--and both are tested independently.

We have demonstrated that genetic effects on gene expression between populations can be calculated by the GS from data with three dimensions: GE level, SNP genotype, and ethnicity. The GS combines two effects of a particular SNP-GE combination--the genotype frequency differences between populations and the differences in the gene expression levels directed by genotypes. Thus, a smaller GS p-value does not necessarily imply a greater allele frequency difference or a substantial SNP effect. To prioritize candidate eSNPs, it is more advantageous to rank the GS among a significant eSNP set rather than to rank their p-values directly. Using this strategy, we demonstrated that a large set of eSNPs contributes to a significant GS, and identified a small subset of 19 unique eSNPs that generated significant absolute GS values (>0.5) between CEU and Asian cohorts. Using this subset of eSNPs, the CEU and Asian cohorts can be easily distinguished. These results indicated that GS not only assisted in filtering functional SNPs but also improved identification of those SNPs with causal potential.

## Conclusions

We investigated the statistical issues associated with common eQTL identification using data combined from different populations. Because differentially expressed genes and SNPs with divergent allele frequencies are common among ethnic populations [47,48], it is believed that combining genetic genomic datasets across populations improves identification of common eQTL. We have demonstrated the impact of MAFs on identification of common eQTL using either separate or combined population data. Quantile transformation to a standard normal distribution is a useful strategy for normalizing gene expression data derived from different populations; however, it did not explicitly take into account the diversity of MAFs among populations and may result in misled statistical conclusions in eQTL study. Instead of transforming ethnic expression data, we showed that application of CTWM to model SNP-GE associations directly onto the original expression data might correct the expression bias arouse from genetic diversity among populations.

We further explored the applicability of CTWM to HapMap populations. As a result, CTWM was shown to be comprehensive and was highly effective in identifying common eQTL in either *local *or *distant *regions with similar SNP effects on GE levels between populations. Further extension of the CTWM to estimate the GS between populations provided an additional statistical test (CTWM-GS method) for identifying eSNPs. This method can also be used to examine genetic genomic data containing both case and control individuals. Such studies can provide new insights into disease etiology by identifying potential eSNPs with allele frequency differences or that are associated with different gene expression levels in the populations.

## Authors' contributions

CLH designed the study, analyzed the data and drafted the paper. IBL helped to develop the methods. ARH participated in the statistical analysis of the results. CSJF provided analytic support and supervised the project. All authors read and approved the final manuscript.

## Supplementary Material

Additional file 1 **Supplementary material**. This PDF contains the details of methods for QT, CTWM, CTWM-GS and simulations. Click here for file

Additional file 2 **Simulations with baseline differences**. This PDF contains the graphs of simulation results at *D *= 1. (A) depicts Type I error (upper panel, E = 0 for d = 0, 0.1, 0.2) and power (lower panel, E = 0.5 for d = 0, 0.1, 0.2) versus different allele frequency of group 0 (*P*_*0*_). The three color bars are as explained in the legend of Figure 1. (B) depicts Type I error rate (E = 0) and power (E = 0.5) versus different allele frequency of group 0 for CTWM-GS. The three color bars are as explained in the legend of Figure 2. Click here for file

Additional file 3 **Estimates of BD and GS**. This PDF summarizes the estimates of BD and GS under the null (upper panel, E = 0) and alternative (lower panel, E = 0.5) hypotheses, respectively, in the simulation studies. The dots are means of the baseline difference (BD, in red) and genetic score (GS, in blue) estimated by CTWM-GS. Arrows of each dot represent the 95% confidence interval calculated from 10,000 simulations. Dash lines are the true values of BD (black) and GS (gray) derived from parameters used in the simulations. Click here for file

Additional file 4 **Summary of putative eQTL generated using CTWM**. This PDF summarizes (A) -log10 p-values or (B) allele frequencies with respect to *local *(blue) and *distant *(red) eQTL by histogram and boxplot underlying the 1,839 putative eQTL identified by CTWM. The histogram is a representation of probability densities (indicated on the x-axis). Click here for file

Additional file 5 **Supplementary table**. This PDF summarizes the 19 eSNPs selected using a GS threshold of 0.5. Click here for file

Additional file 6 **Supplementary simulation results**. This PDF contains the graphs of simulation results as explained in the legend to Figure 1 with three different testing methods as follows: blue, CTWM; yellow, IG method with hypothesis composed of the intersection of 2 sub-hypothesis; brown, two-way ANOVA with interaction term. Click here for file

Additional file 7 **Comparing F test with permutation test**. This PDF describes methods and results for comparing F test with permutation test. Click here for file

Additional file 8 **Results from using tag SNPs**. This PDF summarizes the eQTL data generated by IC and CTWM method, and eSNPs identified by CTWM-GS method underlying 163,448 tag SNPs. Click here for file
