# Supplementary material for: Modeling expression quantitative trait loci in data combining ethnic populations
Source: BMC Bioinformatics. 2010 Feb 27;11:111. doi: 10.1186/1471-2105-11-111 (PMC2844390; doi:10.1186/1471-2105-11-111)
Supplement: Additional file 1 — Supplementary material. This PDF contains the details of methods for QT, CTWM, CTWM-GS and simulations. [file 1471-2105-11-111-S1.PDF]

The QT method

To avoid spurious eQTLs in the data combined from multiple populations due to population structure, Veyrieras et al. (2008) applied a normal quantile transformation (QT) to each gene, within each population before combining data [21]. In application, for each gene, we replace the *r*th biggest of n_i observations in *i*th population by the $((r - 0.5) / n_i)$th quantile of the standard normal distribution. By imposing normality on each GE value in each population, the population effect on the GE difference will be excluded in modeling eQTL. That is, the effect of a SNP on GE levels can be simply modeled by a reparameterization model. When considering $c = 0$ as the baseline genotype, the n (where $n = n_0 + n_1$) signals can be expressed as:

$yt_{ijk} = \mu_{\bullet 0} + \tau_{\bullet j} + e_{ijk}$, where yt_{ijk} is the transformed GE value for the *k*th replication on the *j*th genotype in the *i*th population, and $\mu_{\bullet 0}$ is the expression baseline in the combined data. Examining the associations of SNPs and GE levels can be performed by testing the null hypothesis $H_0 : \tau_{\bullet 1} = \tau_{\bullet 2} = 0$ using the F-statistic calculated from ANOVA.

The constrained two-way model (CTWM) method

Under the CTWM, the n (where $n = n_0 + n_1$) signals can be expressed as $y_{ijk} = \mu_{i0} + \tau_{\bullet j} + e_{ijk}$, where the e_{ijk}'s are assumed to be independently distributed as $N(0, \sigma^2)$, and the regression equation is

$$E(Y) = D\theta \Rightarrow E\begin{pmatrix} Y_{00} \\ Y_{01} \\ Y_{02} \\ Y_{10} \\ Y_{11} \\ Y_{12} \end{pmatrix} = \begin{pmatrix} 1 & 0 & 0 & 0 \\ 1 & 0 & 1 & 0 \\ 1 & 0 & 0 & 1 \\ 0 & 1 & 0 & 0 \\ 0 & 1 & 1 & 0 \\ 0 & 1 & 0 & 1 \end{pmatrix} \times \begin{pmatrix} \mu_{00} \\ \mu_{10} \\ \tau_{\bullet 1} \\ \tau_{\bullet 2} \end{pmatrix}$$

The estimators of the parameter $\theta' = (\mu_{00}, \mu_{10}, \tau_{\bullet 1}, \tau_{\bullet 2})$ can be obtained through the normal equation $(X'X)\theta = X'Y$ in which the design matrix X is generated from D. Because the two independent unbalanced one-way models in the IG approach are combined into a CTWM, the analysis of SNP-GE associations can be performed by testing the null hypothesis $H_0 : \tau_{\bullet 1} = \tau_{\bullet 2} = 0$ using the partial F-statistic:

$F = \dfrac{\left(\mathrm{B}\hat{\theta}\right)' \left[\mathrm{B}(X'X)^{-1}\mathrm{B}'\right]^{-1} \mathrm{B}\hat{\theta} / q}{SSE/\nu}$, where B is in the matrix form $\begin{bmatrix} 0 & 0 & 1 & 0 \\ 0 & 0 & 0 & 1 \end{bmatrix}$, q is the rank of the matrix B, SSE represents the error sum of squares $\sum_i \sum_j \sum_k \left(\mathrm{y}_{\mathrm{ijk}} - \hat{\mathrm{y}}_{\mathrm{ijk}}\right)^2$, with the prediction value $\hat{\mathrm{y}}_{\mathrm{ijk}}$ calculated from the model, and ν represents the degree of freedom for the SSE.

The CTWM-GS method

Although it is possible to solve the normal equation numerically, the method is unable to display the invariant properties of the estimators. To this end, we solve the normal equation described in the CTWM method by maximum likelihood estimation and express the estimators $\hat{\theta}$ using the cell mean as follows:

$\hat{\mu}_{i0} = \left(\bar{y}_{io} M_0 \prod_{t=\{1,2\}} A_t + \sum_{t=\{1,2\}} M_t A_r \left(n_{i0}\bar{y}_{i0} + n_{s0}\bar{y}_{s0} + n_{s0}\left(\bar{y}_{it} - \bar{y}_{st}\right)\right)\right) \Big/ cd$, for i = 0,1

$\hat{\tau}_{\bullet j} = \left(\begin{array}{l} \sum_{i=\{0,1\}} \left(\left(\bar{y}_{ij} - \bar{y}_{i0}\right) A_r n_{i0} n_{ij} \left(n_{s0} + n_{sj}\right)\right) + \\ M_r \left(A_0 \sum_{i=\{0,1\}} n_{ij}\bar{y}_{ij} - A_j \sum_{i=\{0,1\}} n_{i0}\bar{y}_{i0} + \left(n_{00}n_{ij} - n_{10}n_{0j}\right)\left(\bar{y}_{0r} - \bar{y}_{1r}\right)\right) \end{array} \right) \Bigg/ cd$, for j = 1, 2, where $M_j = n_{0j} n_{ij}$; $A_j = n_{oj} + n_{1j}$; $cd = \sum_{j=0}^{2} \left(M_j \prod_{t \neq j} A_t \right)$; s = {0,1} \ {i}, which means s is an element in {0,1} but not in {i}, and r = {0,1,2} \ { j, t}, which means r is an element in {0,1,2} but not in { j, t}.

Consequently, the estimators of GS and BD can be expressed as follows:

$$\hat{BD} = \hat{\mu}_{00} - \hat{\mu}_{10} = \sum_{j=0}^{2} \left(M_j \left(\bar{y}_{0j} - \bar{y}_{1j} \right) \prod_{t \neq j} A_t \right) \Big/ cd$$

$$\hat{GS} = \left(P_{01} - P_{11}\right)\hat{\tau}_{\bullet 1} + \left(P_{02} - P_{12}\right)\hat{\tau}_{\bullet 2}$$

$$= \sum_{j=0}^{2} \left(\left(P_{0j} - P_{1j}\right) \left(\left(M_j \sum_i \bar{y}_{ij} \prod_{t \neq j} n_{it} \right) - \left(\sum_{t \neq j} M_t \sum_i \bar{y}_{it} n_{ij} n_{sr} \right) \right) \right) \Big/ cd$$

On the contrary, the GS in the IG analysis method is represented as *GS* $= \sum_{i=0,1} (-1)^i \sum_{j=1,2} P_{ij} \tau_{ij}$. Applying the constraint $\left(\sum_{j=0,1,2} P_{ij} = 1 \right)$ for each *i*, the estimator of the genetic score is GS = $\left(\bar{y}_{0\bullet\bullet} - \bar{y}_{1\bullet\bullet}\right) - \left(\bar{y}_{00\bullet} - \bar{y}_{10\bullet}\right)$. This quantity cannot be tested directly because the second term in the estimator indicates that the genetic score varies according to the reference genotype chosen.

Simulations

For scenario 1, given an allele frequency difference (d) between populations, SNP genotype data are first simulated. With the given allele frequency of population 0 (P_0), the numbers of individuals carrying distinct genotypes are simulated from a multinomial distribution with parameters $\left(n_0, P_0^2, 2P_0(1-P_0), (1-P_0)^2 \right)$, and the observed allele frequency (P^*) is calculated. For group 1, the genotype frequency is drawn from Hardy-Weinberg proportions calculated by $P_1 = (P^* + d)$ to reach the desired sample size n_1. After genotype data are simulated, the corresponding gene expression data are simulated from N($j \times E$, 1), where $j \in \{0, 1, 2\}$ represents the genotype (0, 1, 2), and E is the magnitude of the SNP effect on the GE. The simulation is repeated 10,000 times with the sample size $n_0 = n_1 = 50$ (similar results were observed for $n_0 = n_1 = 30$ and $n_0 = n_1 = 100$).

For scenario 2, the expression data were simulated from N($i \times D + j \times E$, 1), where index $i \in \{0, 1\}$ represents population 0 and 1, respectively, and D is the baseline difference. We simulated each parameter combination of E = 0, 0.5 and d = 0, 0.2 with D = 1, and compared the results with the simulation using D = 0, in which differential expression was resulted solely from genetic-level differences, as described above.
