# Supplementary figures and images for: Modeling expression quantitative trait loci in data combining ethnic populations
Source: BMC Bioinformatics. 2010 Feb 27;11:111. doi: 10.1186/1471-2105-11-111 (PMC2844390; doi:10.1186/1471-2105-11-111)

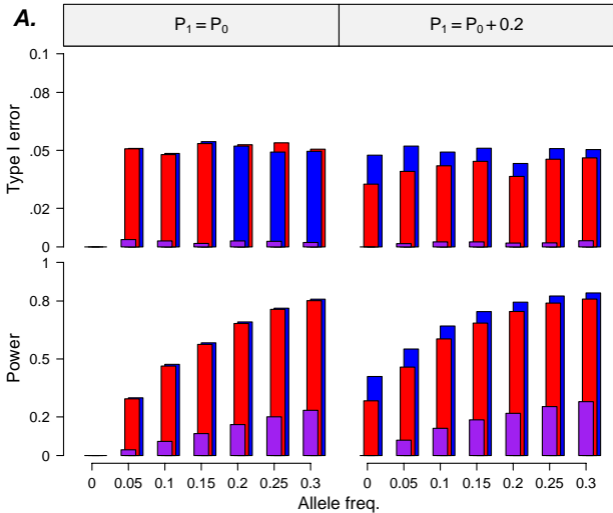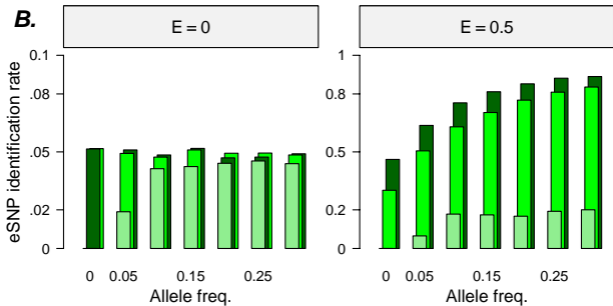

Supplement: Additional file 2 — Simulations with baseline differences. This PDF contains the graphs of simulation results at D = 1. (A) depicts Type I error (upper panel, E = 0 for d = 0, 0.1, 0.2) and power (lower panel, E = 0.5 for d = 0, 0.1, 0.2) versus different allele frequency of group 0 (P 0 ). The three color bars are as explained in the legend of Figure 1. (B) depicts Type I error rate (E = 0) and power (E = 0.5) versus different allele frequency of group 0 for CTWM-GS. The three color bars are as explained in the legend of Figure 2. [file 1471-2105-11-111-S2.PDF]

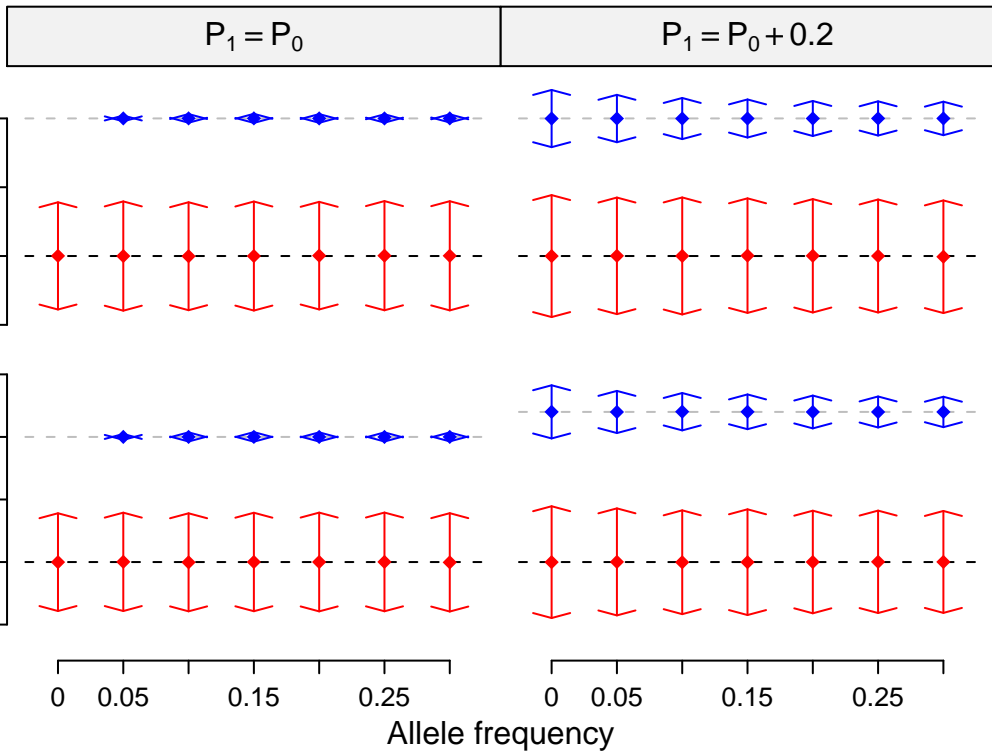

Supplement: Additional file 3 — Estimates of BD and GS. This PDF summarizes the estimates of BD and GS under the null (upper panel, E = 0) and alternative (lower panel, E = 0.5) hypotheses, respectively, in the simulation studies. The dots are means of the baseline difference (BD, in red) and genetic score (GS, in blue) estimated by CTWM-GS. Arrows of each dot represent the 95% confidence interval calculated from 10,000 simulations. Dash lines are the true values of BD (black) and GS (gray) derived from parameters used in the simulations. [file 1471-2105-11-111-S3.PDF]

-Log10 P-value

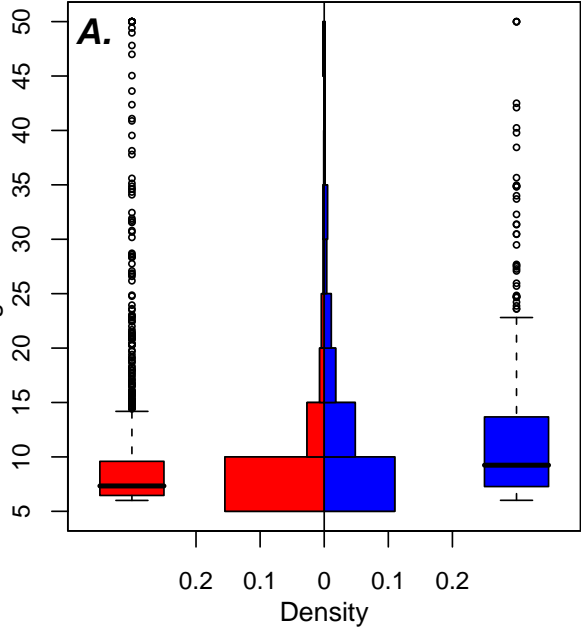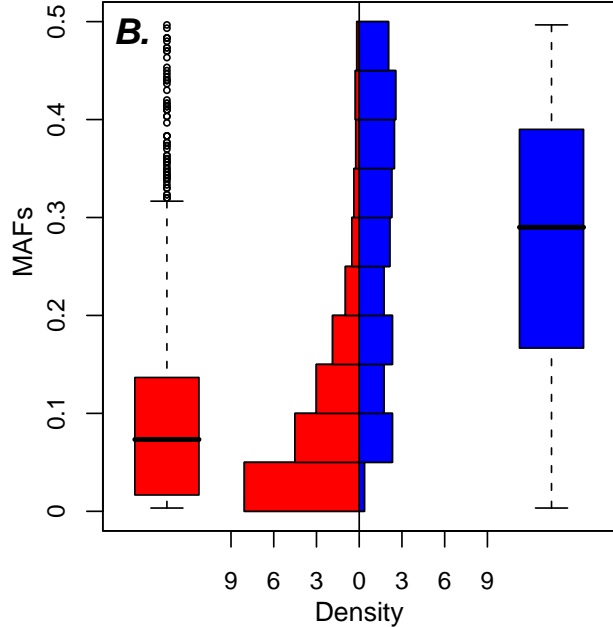

Supplement: Additional file 4 — Summary of putative eQTL generated using CTWM. This PDF summarizes (A) -log10 p-values or (B) allele frequencies with respect to local (blue) and distant (red) eQTL by histogram and boxplot underlying the 1,839 putative eQTL identified by CTWM. The histogram is a representation of probability densities (indicated on the x-axis). [file 1471-2105-11-111-S4.PDF]

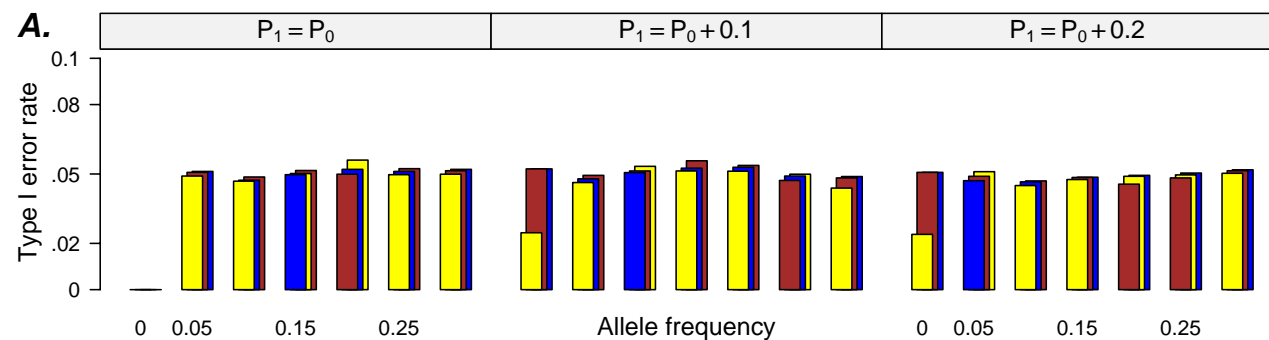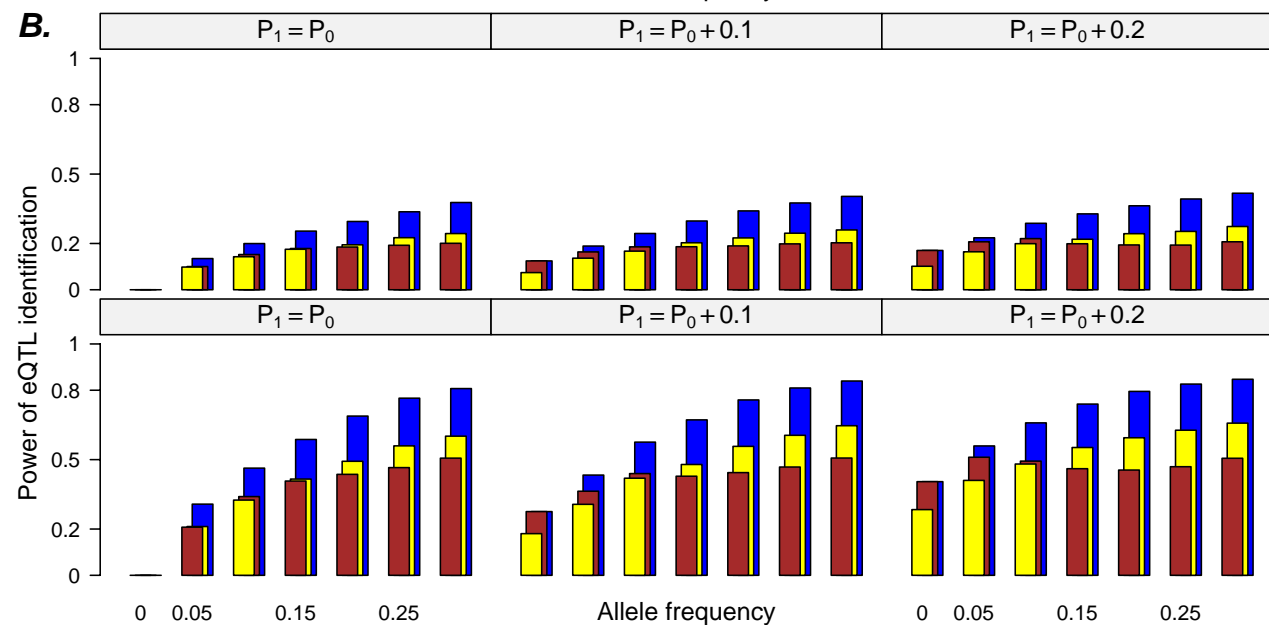

Supplement: Additional file 6 — Supplementary simulation results. This PDF contains the graphs of simulation results as explained in the legend to Figure 1 with three different testing methods as follows: blue, CTWM; yellow, IG method with hypothesis composed of the intersection of 2 sub-hypothesis; brown, two-way ANOVA with interaction term. [file 1471-2105-11-111-S6.PDF]

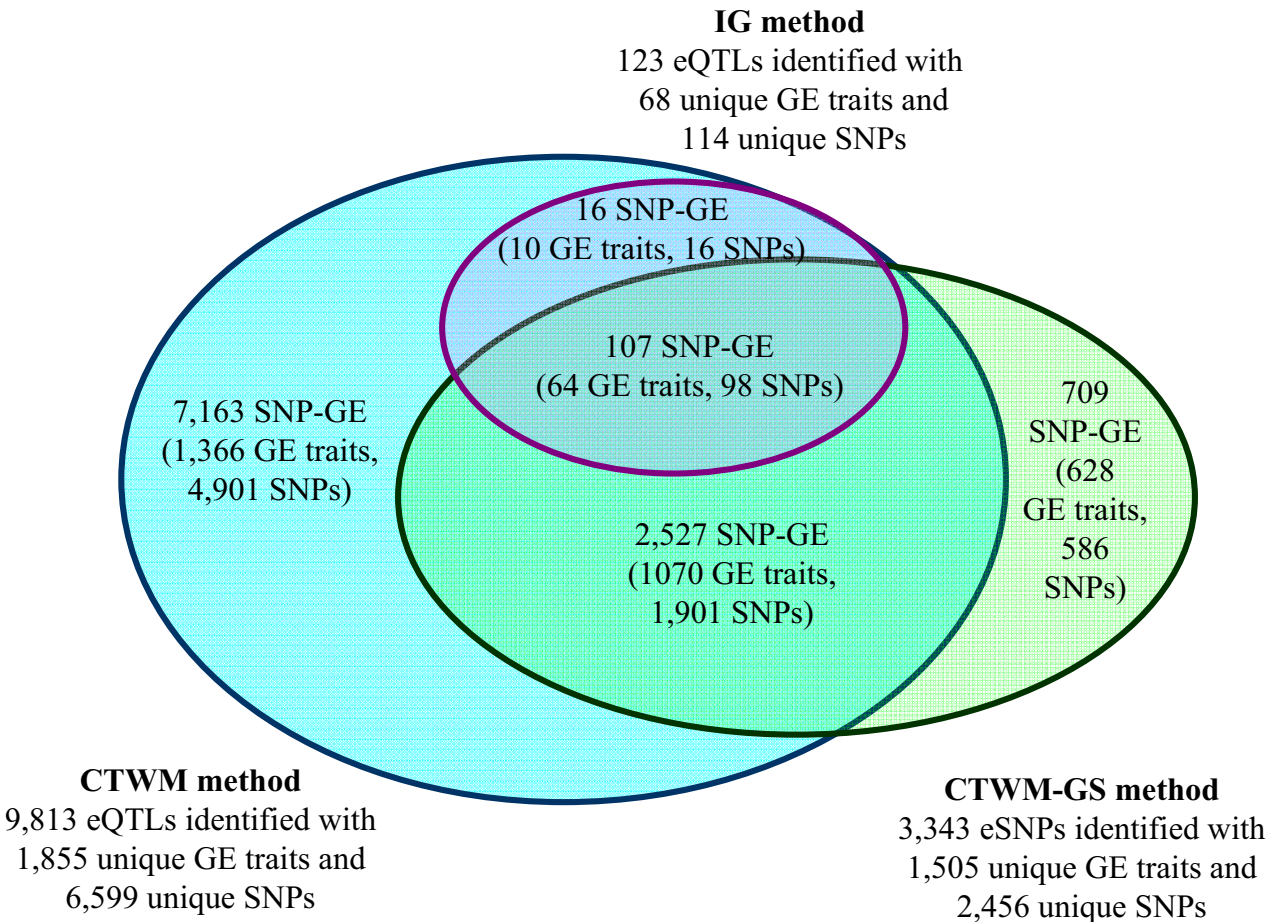

Supplement: Additional file 8 — Results from using tag SNPs. This PDF summarizes the eQTL data generated by IC and CTWM method, and eSNPs identified by CTWM-GS method underlying 163,448 tag SNPs. [file 1471-2105-11-111-S8.PDF]
