# Supplementary material for: Modeling expression quantitative trait loci in data combining ethnic populations
Source: BMC Bioinformatics. 2010 Feb 27;11:111. doi: 10.1186/1471-2105-11-111 (PMC2844390; doi:10.1186/1471-2105-11-111)
Supplement: Additional file 5 — Supplementary table. This PDF summarizes the 19 eSNPs selected using a GS threshold of 0.5. [file 1471-2105-11-111-S5.PDF]

Tables

Table 1 - Summary of eSNP analyses

Gene symbol	eSNP	Allele[1]	MAF[2]		CTWM-GS analysis[3]				IG analysis[4]		Overall mean[5]			
			CEU	Asian	BD	p-value	GS	p-value	CEU	Asian	CEU	Asian	Diff.	p-value
ANKDD1A	rs871447	C/T	0.42	0.03	0.32	1.4E-07	0.67	7.9E-42	9.9E-19	3.8E-18	8.38	7.4	0.99	1.8E-15
FLJ20444	rs41373149	G/C	0.01	0.29	0.26	3.7E-03	-0.72	1.0E-35	1.7E-03	7.0E-31	6.52	6.98	-0.46	1.9E-04
NAPRT1	rs10112966	T/C	0.24	0.57	0.14	1.7E-01	0.75	1.1E-34	2.4E-12	2.4E-22	9.87	8.97	0.9	4.6E-09
HLA-DRB5	rs9271850	A/G	0.18	0.34	0.31	1.7E-01	0.85	5.1E-31	1.6E-08	4.3E-19	13.13	11.96	1.16	2.0E-04
UTS2	rs161822	A/G	0.02	0.53	-0.62	5.5E-05	-1.38	2.3E-25	1.6E-09	3.2E-17	6.54	8.54	-2	1.3E-28
LOC389493	rs11238381	C/G	0.18	0.55	-0.44	1.1E-05	-0.52	9.7E-22	1.3E-11	9.3E-12	6.71	7.66	-0.96	6.6E-14
CHI3L2	rs11102223	T/G	0.51	0.26	-0.2	2.7E-01	-0.69	1.3E-18	1.1E-06	2.2E-17	10.63	11.53	-0.89	4.6E-04
C16orf75	rs4451969	T/C	0.72	0.22	0	9.7E-01	0.52	2.7E-18	2.0E-10	1.5E-07	9.94	9.42	0.52	1.0E-07
C1orf115	rs425437	T/C	0.06	0.32	-0.21	1.3E-01	-0.57	1.5E-16	8.6E-05	5.5E-13	7.07	7.85	-0.78	1.6E-06
C8orf13	rs998683	T/C	0.73	0.33	0.37	1.2E-03	-0.53	6.7E-15	4.4E-06	1.7E-08	7.86	8.02	-0.17	1.5E-01
KIAA0748	rs7962801	T/C	0.89	0.13	0.02	7.8E-01	-0.53	6.6E-14	4.3E-08	5.8E-07	6.87	7.38	-0.51	4.3E-22
MOSC2	rs425437	T/C	0.06	0.32	-0.13	3.8E-01	-0.5	1.2E-11	5.6E-04	9.8E-11	7.69	8.32	-0.63	3.0E-04
TSPAN32	rs756920	C/G	0.06	0.72	0.01	9.3E-01	-0.6	1.5E-10	1.3E-05	1.5E-07	6.39	6.98	-0.59	1.6E-17
IGHA2	rs10483288	A/C	0.22	0.00	-4.27	1.8E-19	1.37	3.5E-09	1.2E-05	NA	10.87	13.77	-2.9	1.3E-09
SYNGR1	rs5757611	T/C	0.24	0.57	-0.05	6.9E-01	-0.51	1.0E-08	1.3E-03	1.0E-05	7.03	7.59	-0.56	8.4E-06
GPER	rs1419772	G/A	0.43	0.02	-0.02	9.0E-01	-0.6	1.3E-08	1.2E-06	1.1E-01	9.5	10.12	-0.62	1.9E-05
C3orf14	rs2337387	T/C	0.59	0.01	-1.71	8.5E-08	1.59	1.5E-08	5.4E-05	7.9E-02	8.7	8.82	-0.12	5.3E-01
IGK	rs604127	T/C	0.19	0.06	-3.39	5.3E-18	0.51	5.9E-07	1.5E-06	7.0E-01	8.03	10.92	-2.89	7.2E-12
IGHV6-1	rs17710010	A/G	0.23	0.00	-1.47	1.9E-11	0.58	8.8E-07	3.0E-04	NA	8.84	9.73	-0.89	4.7E-05
CADM1	rs613699	G/T	0.41	0.00	1.39	1.6E-12	-0.64	9.2E-07	3.6E-04	NA	7.6	6.86	0.74	6.2E-06

[1]The minor/common allele in the combined population. [2]Minor allele frequencies of eSNPs in the CEU and Asian populations. [3]Results generated from CTWM-GS method. The four columns, from left to right, are baseline difference, p-value of the baseline difference tested by the CTWM-GS, genetic score, and p-value of the genetic score tested by CTWM-GS. [4]P-values of the SNP-GE associations in the CEU and Asian populations tested by the IG method. [5]Arithmetic mean of gene expression values in the CEU and Asian populations; the difference of these two means is shown under the column "Diff.", and p-value of the difference is calculated by the Welch's Two-Sample t-test.
