# Supplementary material for: Modeling expression quantitative trait loci in data combining ethnic populations
Source: BMC Bioinformatics. 2010 Feb 27;11:111. doi: 10.1186/1471-2105-11-111 (PMC2844390; doi:10.1186/1471-2105-11-111)

Comparing F test with permutation test

Simulations are performed with E = 0, 0.3, 0.5 when P_0 = 0.2 and d = 0 under simulation scenario 1. For each E value, the simulation is repeated 1,000 times. The permutation tests are performed by randomly permute the SNP genotype codes across individuals, this is because permuting SNP codes can take both SNP-GE association and genotype frequency difference between populations into account simultaneously. For each SNP-GE data, the null hypothesis (H_0: GS = 0) is rejected if the original F value (calculated from the non-permuted data) is larger than 95 percent of the permuted F values (calculated from 1,000 permutation). Results show that permutation approach (green bar) has lower probabilities of rejecting the null hypothesis in comparison with those using non-central F distribution (red bar). In addition, the rejection probabilities of these two methods are both inflated as the E value increases.

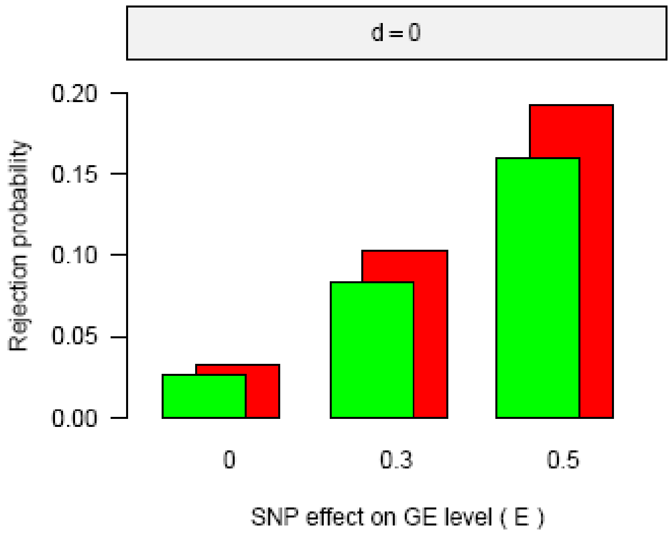

Supplement: Additional file 7 — Comparing F test with permutation test. This PDF describes methods and results for comparing F test with permutation test. [file 1471-2105-11-111-S7.PDF]
